# MiR-29a-3p Improves Acute Lung Injury by Reducing Alveolar Epithelial Cell PANoptosis

**DOI:** 10.14336/AD.2021.1023

**Published:** 2022-06-01

**Authors:** Yanhui Cui, Xueqin Wang, Fengyu Lin, Wen Li, Yuhao Zhao, Fei Zhu, Hang Yang, Mingjun Rao, Yi li, Huaying Liang, Minhui Dai, Ben Liu, Lingli Chen, Duoduo Han, Rongli Lu, Wenzhong Peng, Yan Zhang, Chao Song, Yanwei Luo, Pinhua Pan

**Affiliations:** ^1^Respiratory and critical care medicine, Xiangya Hospital, Central South University, Hunan 410000, China; ^2^Center for neuroscience and behavior, Changsha medical university, Hunan 410219, China; ^3^Infection Control Center, Xiangya Hospital of Central South University, Hunan 410000, China; ^4^Department of Blood transfusion, The third Xiangya Hospital, Central South University, Hunan 410000, China

**Keywords:** acute lung injury, miRNA, PANoptosis, N6-methyladenosine, inflammation

## Abstract

Alveolar epithelial cell damage is an important determinant of the severity of acute lung injury/acute respiratory distress syndrome (ALI/ARDS). However, the molecular mechanisms of alveolar epithelial death during the development of ALI/ARDS remain unclear. In this study, we explore the role of miR-29a-3p in ALI/ARDS and its molecular mechanism. Plasma samples were collected from healthy controls and ARDS patients. Mice were intratracheally instilled with lipopolysaccharide (LPS) to establish acute lung injury. N6-adenosine (m6A) quantification, RNA-binding protein immunoprecipitation, cell viability assay, quantitative real-time polymerase chain reaction, and western blotting were performed. We found that miR-29a-3p was down-regulated in plasma of ARDS patients and lung tissue of ALI model mice, and miR-29a-3p agomir injection down-regulated the levels of the inflammatory factors, including tumor necrosis factor-α (TNF-α), interleukin-1β (IL-1β), and interleukin-6 (IL-6) in the lungs, reducing alveolar epithelial cell PANoptosis as evaluated by the downregulation of Z-DNA binding protein 1 (ZBP1), gasdermin D (GSDMD), caspase-3, caspase-8, and mixed lineage kinase domain-like protein (MLKL), ultimately improving lung injury in the ALI model mice. Mechanism studies demonstrated that the knockout of methyltransferase 3 (N6-adenosine-methyltransferase complex catalytic subunit) removed the m6A modification of miR-29a-3p and reduced miR-29a-3p expression. Our findings suggest that miR-29a-3p is a potential target that can be manipulated for ALI/ARDS.

Approximately 1 in 10 patients in the intensive care unit will suffer from acute lung injury/acute respiratory distress syndrome (ALI/ARDS), which has a high mortality rate [1]. The pathogenic factors of ALI/ARDS include direct injury and indirect injury, both of which can cause alveolar epithelial cell damage or death and aggravate alveolar inflammation [2, 3]. Alveolar epithelial cell damage is an important determinant of the severity of ARDS [4, 5]. Therefore, it is critical to understand the molecular mechanisms of alveolar epithelial injury and excessive inflammation in the process of ALI/ARDS.

MicroRNAs (miRNAs) are a type of endogenous non-coding single-stranded RNA with a length of approximately 22 nucleotides that inhibit gene expression by binding to the 3'UTR sequence of the target gene. In recent years, the role of miRNA in the occurrence and development of ALI/ARDS has been extensively studied [6, 7]. MiR-29a-3p is a member of the miR-29 family that plays an important regulatory role in immune regulation and inflammation [8-10]. Transfection of miR-29a-3p mimics into CD4+ T cells can significantly increase the ratio of Treg/Th17 cells [11]. Tail vein injection of miR-29a-3p nanoparticles can prevent and treat mouse colitis by inhibiting the interferon-related inflammatory cascade [12]. Wang et al. found that resveratrol up-regulated the expression of miR-29a, thereby activating the nuclear factor E2-related factor 2 signaling pathway, inhibiting the production of intracellular reactive oxygen species and the proliferation of fibroblast-like synovial cells, and improving rat rheumatoid arthritis [13]. Tian et al. found that miR-29a-3p targeted to cyclin T2 to reduce serum inflammation and cardiomyocyte apoptosis, and improve myocardial ischemia-reperfusion injury [14]. However, the role of miR-29a-3p in the occurrence and development of ALI/ARDS is unclear.

N6-methyladenosine (m6A) modification is a common modification that regulates the function of miRNA [15, 16]. Alarcón et al. found that methyl-transferase 3 (METTL3, the core methyltransferase that mediates m6A modification) dependent m6A modification is necessary for the cleavage and processing of most pri-miRNAs into mature miRNAs [17]. Knockout of METTL3 resulted in an approximately 70% reduction in mature miRNA levels, while overexpression of METTL3 increased mature miRNA levels [17]. However, it is unclear whether the METTL3-dependent m6A modification regulates the function of miR-29a-3p during the development of ARDS.

In this study, we explore the role of miR-29a-3p in ALI/ARDS and its molecular mechanism. We found that miR-29a-3p was down-regulated in plasma of ARDS patients and the lung tissue of ALI model mice, and miR-29a-3p agomir injection down-regulated the levels of inflammatory factors in the lungs, reducing PANoptosis alveolar epithelial cells, and ultimately improving lung injury in the ALI model mice. PANoptosis is a programmed cell death that assembles pyroptosis, apoptosis, and necroptosis pathways, and this highlights the crosstalk and coordination of these three pathways [18]. Mechanism studies found that METTL3 enhanced m6A modification of miR-29a-3p and increased miR-29a-3p expression. Our findings suggest that miR-29a is a potential target that can be manipulated for ALI/ARDS.

## MATERIALS AND METHODS

### Human subjects

The human sample collection was approved by the ethics committees of the Xiangya Hospital (No. 202004119). Written forms of consent were obtained from all subjects. Consistent with previous studies [19], all subjects had ARDS according to the current Berlin definition: (1) acute onset of dyspnoea with a history of aspiration within one week and incubation for mechanical ventilation support; (2) partial pressure of oxygen (PaO2)/FiO2 equal to or less than 300 mmHg over 48 h; and (3) new infiltration shadow on chest imaging. Patients who had sepsis, lung fibrosis, or pregnant were excluded. Fifteen ARDS patients were included. Fifteen age- and sex-matched healthy volunteers were enrolled as controls. The patient's comorbidities and the cause of ARDS are shown in Table 1. Peripheral blood samples were collected in tubes containing anticoagulant (EDTA) within 24 hours of the diagnosis of ARDS. The samples were centrifuged at 3000 rpm for 10 min for plasma collection, then stored at -80°C. To measure the level of miR-29a-3p, plasma miRNA was extracted using a commercial kit (Qiagen, 217184), and reverse transcription was performed using the All-in-One™ miRNA First-Strand cDNA Synthesis Kit (GeneCopoeia, QP013). miR-29a-3p (GeneCopoeia, HmiRQP0371) and U6 (GeneCopoeia, HmiRQP9001) expressions were examined using SYBR green fluorescent quantitative real-time PCR (GeneCopoeia, QP010).

### ALI model and treatment

Eight-week-old male C57BL/6 mice obtained from the Laboratory Animal Center of Central South University (Hunan, China) were used in this study. The mice were housed (5 mice per cage) in a temperature-controlled (24 ± 2 ?) specific-pathogen-free facility under 12-h light/dark cycles with free access to food and water. Animal experiments were approved by the Animal Care and Use Committee of Central South University (No. 2020sydw0218). The mice were acclimatized to the environment for at least 1 week before the experiments. All the mice were randomly divided into three groups: the normal saline group (NS), the lipopolysaccharide + negative control group (LPS + NC), and the LPS + miR-29a-3p agomir group (LPS + Agomir). The mice were anesthetized using sodium pentobarbital (50 mg/kg i.p.) and then intratracheally instilled with LPS (Sigma, L2630, 5 mg/kg) + oligonucleotide (NC or agomir provided by Genepharma, 10 nmol per mouse) or NS. Twelve hours after the intratracheal injection of LPS, the mice were euthanized using an over dose of sodium pentobarbital (100 mg/kg i.p.) to obtain the tissues for further analysis.

**Table 1:** Clinical characters of ARDS patients.

ARDS patient	Age (years)	Gender	Diabetes	Hypertension	Smoking history	Pathogen	APACHE II	PaO2/FiO2
1	26	Male	No	No	Yes	Virus	16	177
2	38	Male	No	No	Yes	Virus	6	245
3	68	Female	No	No	No	Bacteria	13	90
4	54	Male	Yes	Yes	Yes	Bacteria	14	91
5	41	Male	No	No	No	Bacteria	16	180
6	62	Female	No	Yes	No	Bacteria	12	99
7	25	Female	No	No	No	Virus	7	240
8	69	Male	No	Yes	No	Bacteria	11	245
9	50	Female	No	No	No	Bacteria	16	230
10	40	Male	No	No	Yes	Bacteria	14	150
11	30	Female	No	No	No	Bacteria	14	96
12	33	Male	No	No	Yes	Bacteria	4	241
13	62	Male	Yes	No	Yes	Virus	3	214
14	42	Female	No	Yes	No	Virus	13	178
15	68	Female	No	No	No	Virus	16	150

### Cell culture and transfection

Human alveolar epithelial cells (A549 Cells) were purchased from ATCC and were maintained in Dulbecco’s Modified Eagle Medium supplemented with 10 % fetal bovine serum, and a penicillin-streptomycin solution in a humidified incubator with 5% CO_2_ at 37 °C. The siRNA of METTL3 (5'-CTGCAAGTATGTTCA CTATGA-3'), and tumor necrosis factor receptor 1 (TNFR1) (Santa Cruz, sc-29507) were transfected into cells using Lipofectamine 2000. The miR-29a-3p agomir were transfected into cells using GP-transfect-Mate (Genepharma, G04008).

### Hematoxylin-eosin (HE) staining

Lung tissues were fixed in 4% polyformaldehyde (Servicebio, G1101) and then dehydrated and embedded in paraffin. Tissue sections were cut and subjected to HE staining.

### In situ hybridization (ISH)

For the situ hybridization, tissues were fixed in 4% polyformaldehyde (containing 1‰ diethypyrocarbonate) for less than 2 hours. The expression of miR-29a-3p was determined using the ISH kit (Boster, MK10282) according to the manufacturer’s instruction.

### Collection and analysis of BALF

The BALF was collected using a syringe with 1 ml of ice-cold phosphate buffer solution (PBS) intratracheally instilled into the lung and then lavaged three times. The bronchoalveolar lavage fluid (BALF) was centrifuged at 1500?rpm for 10?min, and the BALF supernatant was stored at -?80?°C for further detection. The BALF protein concentrations were determined using the bicinchoninic acid (BCA) protein assay kit (Beyotime, P0010). Lactate dehydrogenase (LDH) activity in the BALF was determined using an LDH Cytotoxicity Assay Kit (Nanjing Jiancheng Bioengineering Institute, A020-2-2). Mouse cytokine enzyme-linked immunoassay (ELISA) kits (Cusabio) were used to measure the levels of tumour necrosis factor α (TNF-α), interleukin 1β (IL-1β) and interleukin 6 (IL-6) according to the manufacturer's instructions.

### Lung wet/dry ratio

The lung tissues were weighed and recorded as the wet weight (W) after collection. The tissues were then dehydrated at 65 °C for 48 h and weighed again to obtain the dry weight (D).

### m6A quantification

The total RNA was extracted from the lung tissues using Trizol reagent (Thermo, cat no. 15596026), and then the m6A methylated RNA was quantified using the EpiQuik m6A RNA methylation quantification kit (Epigentek, P-9005) according to the manufacturer's instructions.

### RNA-binding protein immunoprecipitation (RIP)

A Magna RIP™ RNA-Binding Protein Immuno-precipitation Kit (Millipore, cat no. 17-700) was used for RNA immunoprecipitation according to the manufacturer's instructions. Briefly, a single cell suspension of lung tissue was prepared in ice-cold PBS, and lysed with RIP lysis buffer on ice for 15?min. After centrifugation, the supernatant was incubated with protein A/G magnetic beads and m6A antibody (Abcam, ab151230) at 4°C overnight. The immunoprecipitated RNA was then analyzed using RT-qPCR.

### Western blotting

The total protein was extracted using a total protein extraction buffer premixed with protease inhibitor (Thermo) and quantified using a BCA protein assay kit. Equal amounts of protein (25 μg) were separated by 10% or 12% sodium dodecyl sulfate-polyacrylamide gel electrophoresis and transferred onto a polyvinylidene fluoride membrane. The membrane was blocked with 5% fresh nonfat milk in tris buffered saline (containing 0.5‰ tween-20, TBST) at room temperature for 1h and then incubated with antibodies against METTL3 (Affinity, DF12020, 1:1000), TNFR1 (Proteintech, 21574-1-AP, 1:1000), Caspase-3 (Proteintech, 19677-1-AP, 1:1000), Caspase-8 (Proteintech, 13423-1-AP, 1:1000), GSDMD (Abclonal, A18121, 1:1000), MLKL (Proteintech, 66675-1-Ig, 1:2000), ZBP1 (Adipogen, AG-20B-0010, 1:1000) overnight at 4 °C. After three washes with TBST, the membranes were incubated with secondary antibody (Li-cor) for 1 h at room temperature. The membranes were then visualized using the LI-COR Odyssey Scanning Imager.

**Figure 1.**
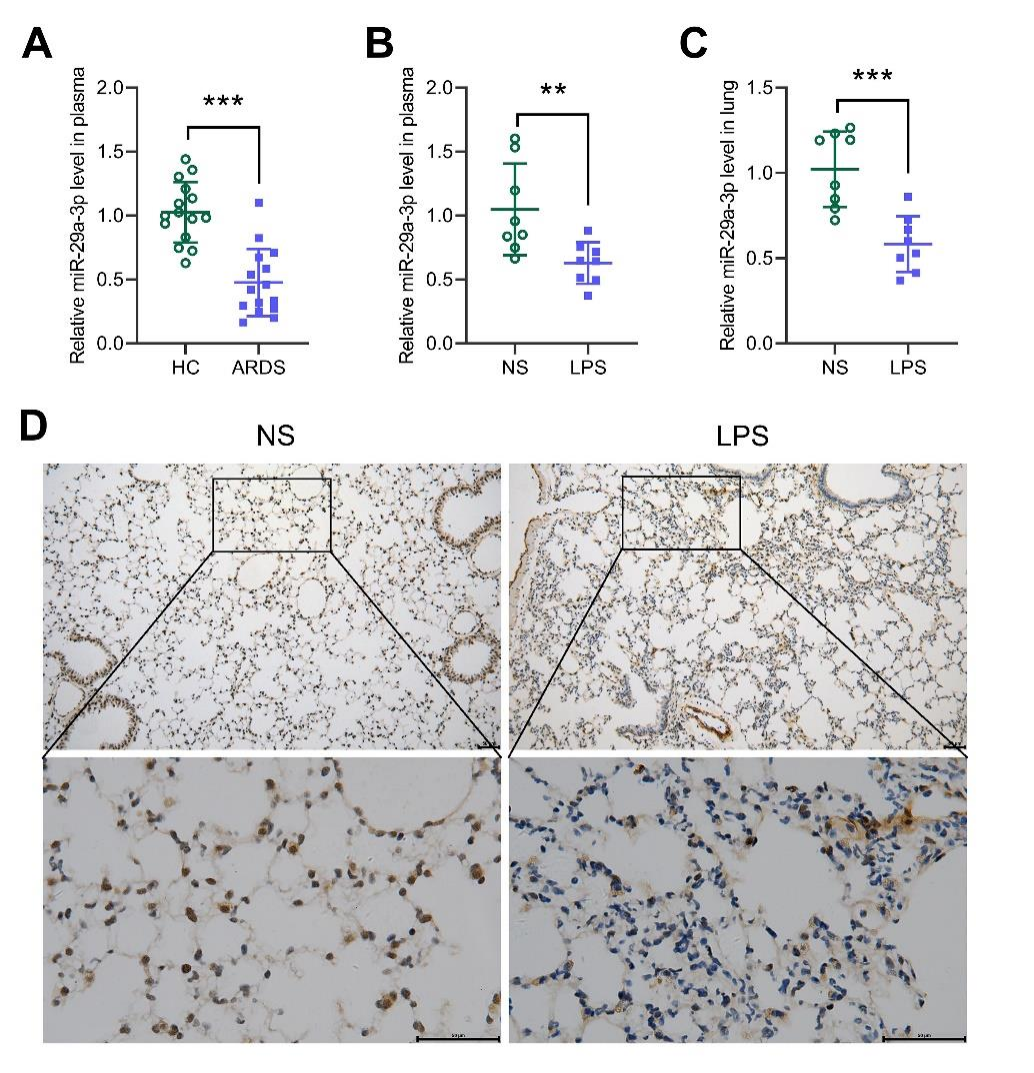
MiR-29a-3p is down-regulated in the plasma of ARDS patients and lung tissue of ALI model mice. (A) RT-qPCR analysis for miR-29a-3p expression in the plasma of healthy controls (N=15) and ARDS patients (N=15). HC, healthy control. ARDS, acute respiratory distress syndrome. (B) RT-qPCR analysis for miR-29a-3p expression in the plasma of ALI mouse model. (C) RT-qPCR analysis for miR-29a-3p expression in the lung tissue of ALI mouse model. (D) In situ hybridization analysis for miR-29a-3p expression in the lung tissue of ALI mouse model. scale bar = 50 μm. NS, normal saline. LPS, Lipopolysaccharide. *** *p* <0.001.

### RT-qPCR

The total RNA was extracted using Trizol reagent, and reverse-transcribed into cDNA using the All-in-One™ miRNA First-Strand cDNA Synthesis Kit (GeneCopoeia, QP013) or HiScript II Q RT SuperMix for qPCR (Vazyme, R223). cDNA amplification was performed using the All-in-One™ miRNA qPCR Kit (GeneCopoeia, QP010) or the ChamQ Universal SYBR qPCR Master Mix (Vazyme, Q711) according to the manufacturer’s instructions on a QuantStudio 3 real-time PCR system. The primers used for the RT-qPCR are listed in Table 2. The comparative Ct method (2^-ΔΔCt^) was used to analyze the data.

### Cell viability assay

Cell viability was investigated using the CCK-8 kit (Beyotime, C0041). Approximately 3000 cells were inoculated into a 96-well plate and incubated overnight. After the indicated treatments, the cells were incubated with the CCK-8 solution, and the absorbance value of each well was measured at 450?nm using a microplate reader.

### Statistical analysis

The statistical analyses were conducted using Graph Pad Prism 8 software. The data are presented as the mean ± SD. A two-tailed Student’s t-test was used to assess the differences between the two groups, and one-way ANOVA coupled with the Sidak’s post-hoc test was used to assess differences among the three groups. *P* < 0.05 was statistically significant.

**Figure 2.**
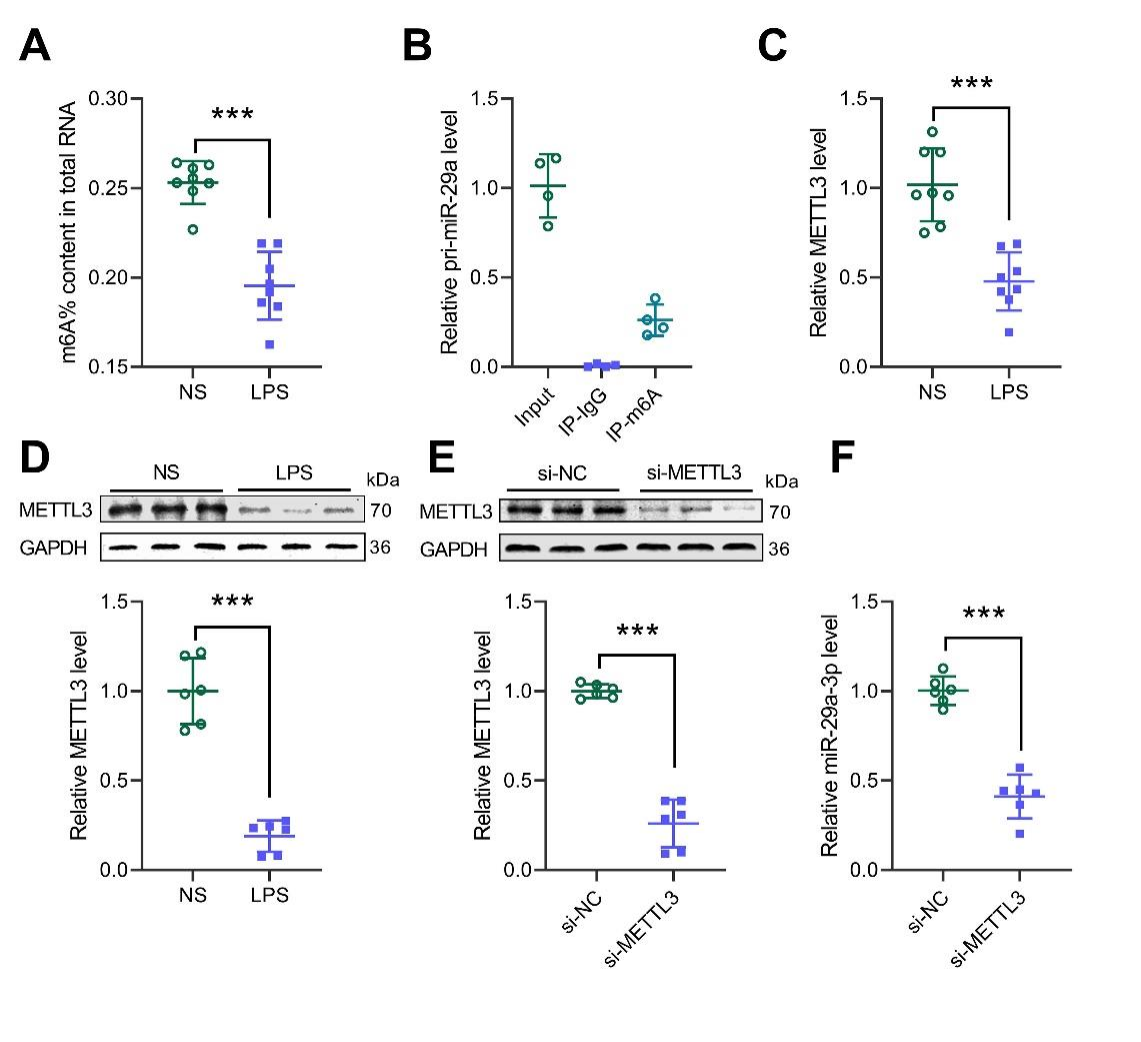
METTL3-mediated m6A modification stabilizes the expression of miR-29a-3p. (A) The level of total m6A in the lung tissue of ALI mouse model. (B) m6A RIP detects the m6A level of pri-miR-29a in the lung tissue of ALI mouse model. (C) RT-qPCR detection of METL3 expression in the lung tissue of ALI mouse model. (D) Western blot analysis for the expression of METTL3 in the lung tissue of ALI mouse model. (E) Western blot analysis for the expression of METTL3 in A549 cells after siRNA transfection. (F) RT-qPCR analysis for the level of miR-29a-3p in A549 cells after METTL3 siRNA transfection. *** *p* <0.001.

**Figure 3.**
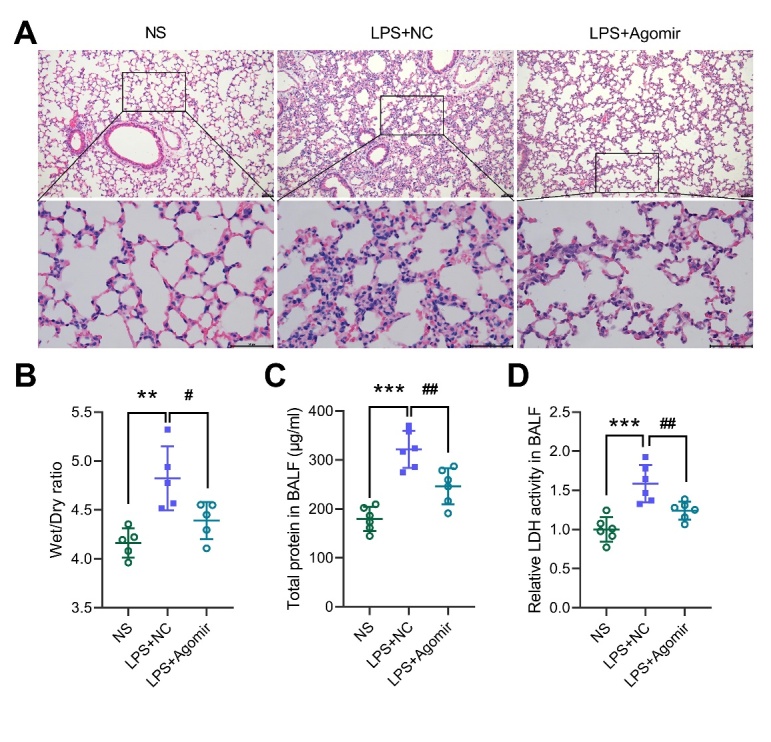
miR-29a-3p improves lung injury in ALI mice. (A) HE staining of lung tissue. scale bar = 50 μm. (B) Lung tissue wet/dry ratio. (C) Total protein concentration in the BALF. (D) LDH activity in the BALF. ** *p* <0.01, *** *p* <0.001 vs NS; # *p* <0.05, ## *p* <0.01 vs LPS+NC.

## RESULTS

### MiR-29a-3p was down-regulated in the plasma of ARDS patients and lung tissue of ALI model mice

We detected the expression of miR-29a-3p in the plasma of 15 patients with ARDS by RT-qPCR. The expression level of miR-29a-3p in the ARDS patients was significantly lower than that of the healthy controls (Fig. 1A). In addition, we found that miR-29a-3p expression was negatively correlated with the TNF-α plasma levels and the number of leukocytes in ARDS patients (Supplementary Fig. 1A, B). We further constructed an ALI mouse model and detected the expression of miR-29a-3p in mouse plasma and lung tissue using RT-qPCR and in situ hybridization. Compared with the NS group, the expression of miR-29a-3p in the plasma (Fig. 1B) and lung tissue of the ALI mice was significantly down-regulated (Fig. 1C-D), and the expression of miR-29a-3p in the plasma of ALI mice were negatively correlated with the TNF-α plasma levels (Supplementary Fig. 1C).

### METTL3-mediated m6A modification stabilizes the expression of miR-29a-3p

Studies have shown that m6A modification plays an important role in miRNA maturation. We wanted to know whether the expression of miR-29a-3p was regulated by m6A modification. Compared with the normal control group, the total m6A level in the lung tissue of the ALI mice was significantly reduced (Fig. 2A). By using the m6A RIP analysis, it was found that pri-miR-29a was modified by m6A (Fig. 2B). In addition, the expression of m6A methyltransferase METTL3 in the lung tissue of the ALI mice was significantly down-regulated compared with the NS group (Fig. 2C-D). Knockdown of METTL3 in the A549 cells significantly reduced the expression of miR-29a-3p (Fig. 2E-F), suggesting that METTL3 enhances the expression of miR-29a-3p by mediating m6A modification in pri-miR-29a-3p.

**Figure 4.**
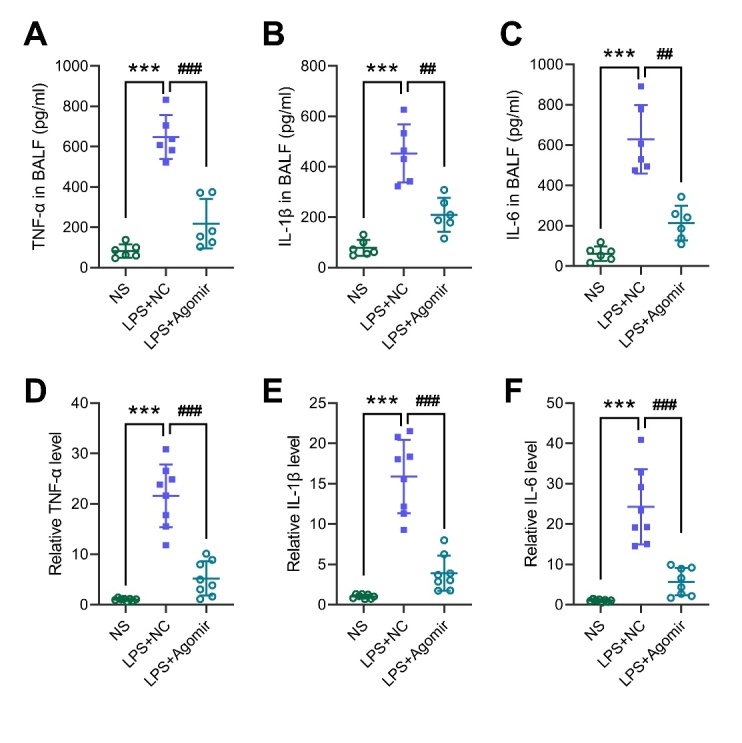
miR-29a-3p agomir reduces lung inflammation in ALI mice. (A-C) ELISA assays were performed to measure the levels of TNF-α, IL-1β, and IL-6 in the BALF after LPS or combined miR-29a-3p agomir treatment. (D-E) RT-qPCR analysis for the mRNA levels of TNF-α, IL-1β, and IL-6 in the lung tissues after LPS or combined miR-29a-3p agomir treatment. *** *p* < 0.001 vs NS; ## *p* < 0.01, ### *p* < 0.001 vs LPS+NC.

### miR-29a-3p improves lung injury in ALI mice

To further explore the role of miR-29a-3p in mouse ALI, we injected miR-29a-3p agomir into the ALI mice to observe the lung injury. HE staining showed that the lung tissue of the LPS+NC group had obvious morphological damage, including inflammatory cell infiltration, and thickening of the alveolar septum, and this damage was significantly reduced in the miR-29a-3p agomir group (Fig. 3A). The LPS-induced lung wet/dry ratio was partially reversed by the miR-29a-3p agomir treatment (Fig. 3B). The LPS increased the total protein and the LDH content in the BALF, whereas the miR-29a-3p agomir treatment could reduce their levels (Fig. 3C-D), suggesting that increasing miR-29a-3p expression can significantly improve lung injury in ALI mice.

### miR-29a-3p agomir reduces lung inflammation in ALI mice

ALI/ARDS is characterized by excessive inflammation. We detected the levels of inflammatory factors, TNF-α, IL-1β, and IL-6, in the BALF using ELISA kits. The results showed that the levels of TNF-α, IL-1β and IL-6 in the LPS+NC group were significantly up-regulated. The miR-29a-3p agomir treatment can reduce the protein levels of inflammatory factors in the BALF (Fig. 4A-C) and downregulated their mRNA levels in the lung tissues of ALI mice (Fig. 4D-F). This result suggested that miR-29a-3p improved lung injury by inhibiting inflammation in ALI mice.

### miR-29a-3p targets TNFR1 to inhibit PANoptosis in alveolar epithelial cells

**Figure 5.**
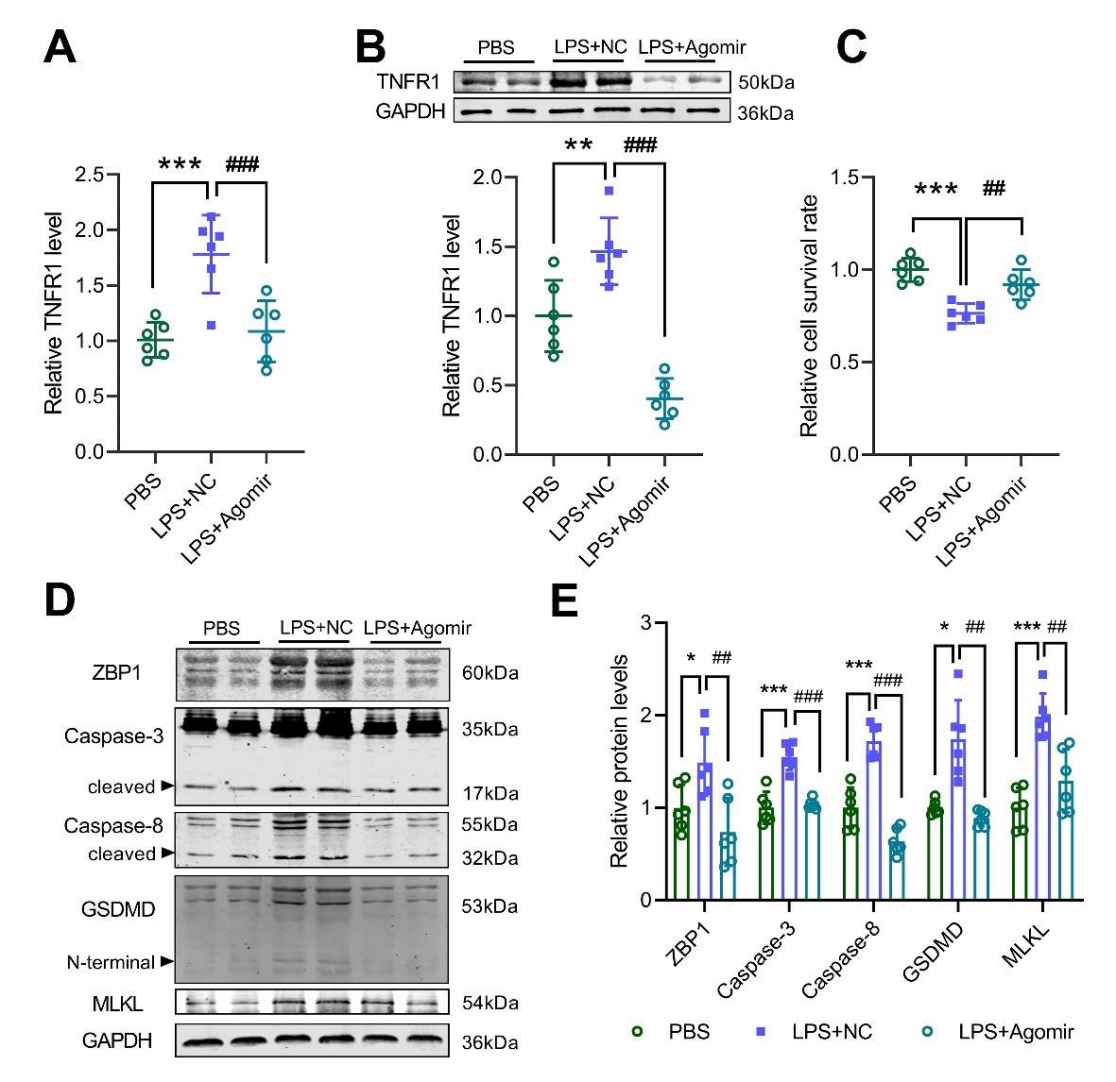
miR-29a-3p targets TNFR1 to inhibit PANoptosis in alveolar epithelial cells. (A) RT-qPCR analysis for the expression of TNFR1 in A549 cells after LPS or combined miR-29a-3p agomir treatment. (B) Western blot analysis for the expression of TNFR1 in A549 cells after LPS or combined miR-29a-3p agomir treatment. (C) CCK8 assay was performed to measure the cell viability of A549 cells after LPS or combined miR-29a-3p agomir treatment. (D-E) Western blot analysis for the expression of PANoptosis markers in A549 cells after LPS or combined miR-29a-3p agomir treatment (D), and quantification (E). * *p* <0.05, *** *p* <0.001 vs NS; ## *p* <0.01, ### *p* <0.001 vs LPS+NC.

**Figure 6.**
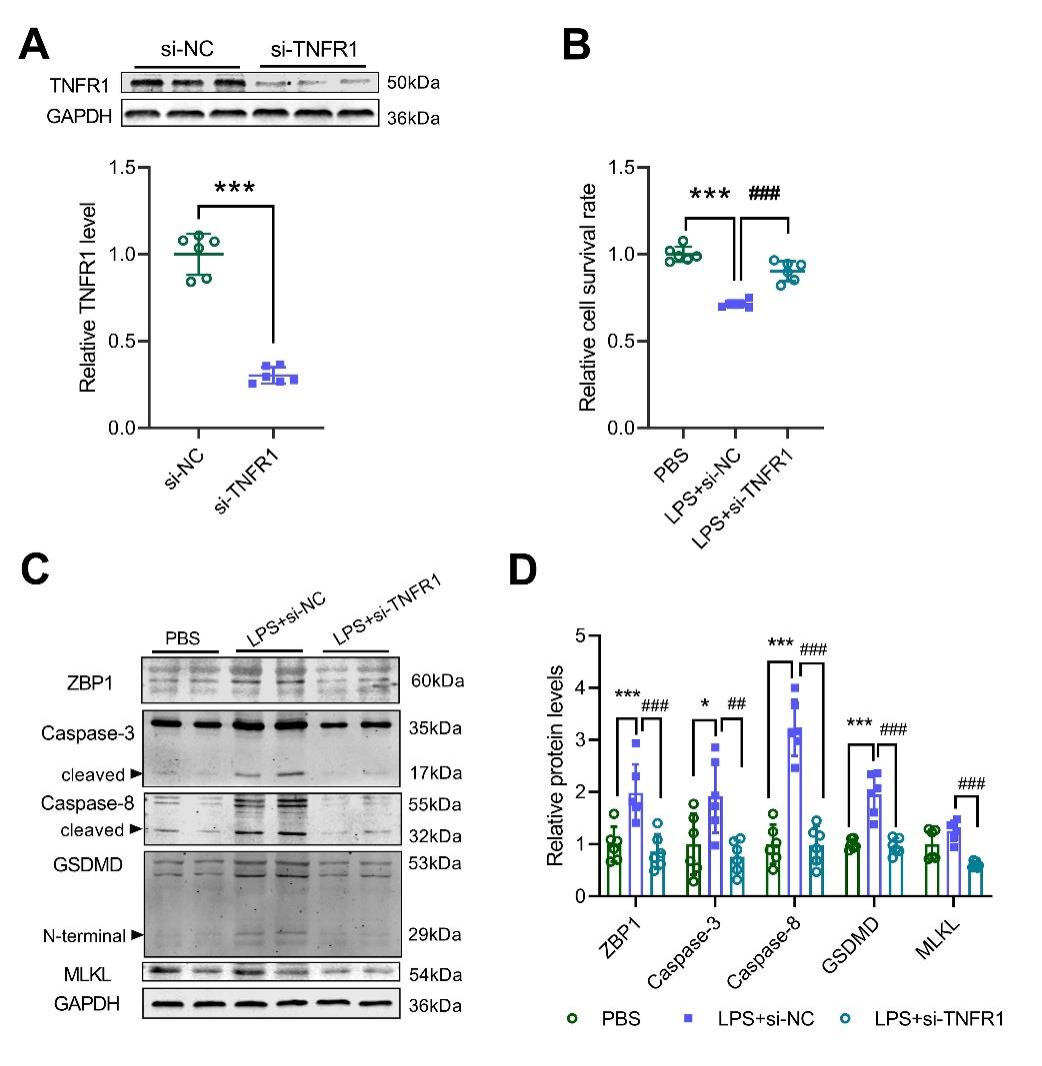
TNFR1 knockdown inhibits PANoptosis in alveolar epithelial cells. (A) Western blot analysis for the expression of TNFR1 in A549 cells after siRNA transfection. (B) CCK8 assay was performed to measure the cell viability of A549 cells after LPS or combined with TNFR1 siRNA treatment. (C-D) Western blot analysis for the expression of PANoptosis markers in A549 cells after LPS or combined TNFR1 siRNA treatment (D), and quantification (E). * *p* <0.05, *** *p* <0.001 vs NS; ## *p* <0.01, ### *p* <0.001 vs LPS+NC.

TNFR1 is the target gene of miR-29a-3p [20], and it is closely related to inflammation, apoptosis, and necrosis [21, 22]. PANoptosis is a form of inflammatory cell death that is highly associated with the activation of the death complex PANoptosome [23]. Therefore, the relationship between miR-29a-3p and TNFR1 may be closely related to PANoptosis. We found that LPS treatment significantly increased TNFR1 mRNA and protein levels, while miR-29a-3p agomir treatment reduced TNFR1 expression (Fig. 5A-B). In addition, miR-29a-3p agomir treatment reduced A549 cell death caused by LPS stimulation (Fig. 5C). Furthermore, we detected the expression of PANoptosis markers, ZBP1 (the key “switch” of PANoptosis), GSDMD (one of the pyroptosis executive proteins), and caspase-3/caspase-8 (one of the apoptosis executive proteins), and MLKL (cell necrosis executive protein) expression. The results showed that LPS stimulation increased the expression of ZBP1, GSDMD, caspase-3, caspase-8, and MLKL in A549 cells, while miR-29a-3p agomir treatment down-regulated the expression of these proteins (Fig. 5D-E). In addition, TNFR1 siRNA transfection reduced the expression of TNFR1 (Fig. 6A), reversed the cell death caused by LPS (Fig. 6B), and reduced the expression levels of ZBP1, GSDMD, caspase-3, and MLKL (Fig. 6C-D). These data indicated that miR-29a-3p targets TNFR1 to inhibit PANoptosis in alveolar epithelial cells.

## DISCUSSION

In this study, we demonstrated for the first time that miR-29a-3p plays an important role in the development of ALI/ARDS, which is a type of clinical syndrome caused by inflammatory response disorders. Our results showed that miR-29a-3p agomir treatment reduced the level of inflammatory factors in lung tissue and improved lung injury in ALI mice.

We found that METTL3-mediated m6A modification increased the expression of miR-29a-3p. m6A is the methylation that occurs on the sixth nitrogen atom (N) of the base adenylate (A), which is the most common post-transcriptional modification of RNA in eukaryotes, and it plays an important regulatory role in RNA metabolism, including translation, splicing, export, and degradation [24]. m6A modification is a dynamic and reversible process consisting of a series of enzymatic reactions: methyltransferase complexes catalyze m6A modification; demethylases remove m6A modification; and methylation readers recognize and bind m6A modification, and mediate the corresponding RNA metabolism process [24]. Studies have shown the role of m6A in inflammation regulation. One previous study demonstrated that m6A-XPO1-NFκB activation leads to intestinal inflammation, and this was discovered to be a new pathological mechanism of coeliac disease [25]. It has also been shown that m6A modification of PPAR-α activates NLRP3 inflammasomes and NF-κB-driven kidney inflammation [26]. Moreover, the crosstalk between m6A and H3K27 trimethylation during bacterial infection regulates the expression of inflammatory factors[27]. We found that the level of m6A in the lung tissue of ALI mice was significantly reduced, suggesting that it may play a role in the process of ALI/ARDS disease. Thus, the role of m6A modification in ARDS warrants further study.

PANoptosis is a form of cell death that is highly connected to pyrolysis, apoptosis, and necrosis. A previous study demonstrated that miR-29a-3p targeted to TNFR1 [20]. Our study also confirmed that miR-29a-3p inhibited the expression of TNFR1. TNFR1 is a cell death receptor, that can activate caspase-3 and caspase-8 after binding to the ligand TNF and promote cell death[28]. Caspase-8 can regulate the inflammatory pathway downstream of ZBP1 and the cellular necroptosis pathway mediated by MLKL [29]. Caspase-8 can also induce the lysis of GSDMD and GSDME to promote pyrolysis [30-32]. Alveolar epithelial cell injury or death is a key feature of ALI/ARDS disease progression. Our study found that LPS stimulation up-regulated the expression of PANoptosis-related molecules in alveolar epithelial cells, and miR-29a-3p agomir or TNFR1 siRNA treatment down-regulated the expression of these molecules and increased cell survival. Clinical trials found that GSK1995057, a specific antagonist of TNFR1, could significantly reduce the pulmonary inflammation of experimental ALI[33]. Therefore, we speculate that miR-29a-3p may inhibit alveolar epithelial cell PANoptosis by targeting TNFR1.

## Conclusion

m6A modification mediated by METTL3 can stabilize the expression of miR-29a-3p. miR-29a-3p inhibited PANoptosis and inflammation in alveolar epithelial cells by targeting TNFR1. Our research indicates that miR-29a-3p may be a potential target that can be manipulated for ALI/ARDS treatment.

## Supplementary Materials

The Supplementary data can be found online at: www.aginganddisease.org/EN/10.14336/AD.2021.1023.


