# Supplementary material for: MiR-29a-3p Improves Acute Lung Injury by Reducing Alveolar Epithelial Cell PANoptosis
Source: Aging Dis. 2022 Jun 1;13(3):899–909. doi: 10.14336/AD.2021.1023 (PMC9116916; doi:10.14336/AD.2021.1023)
Supplement: Supplementary file 1 [file AD-13-3-899-s.pdf]

SUPPLEMENTARY DATA

MiR-29a-3p Improves Acute Lung Injury by Reducing Alveolar Epithelial Cell PANoptosis

Yanhui Cui[1], Xueqin Wang[2], Fengyu Lin[1], Wen Li[1], Yuhao Zhao[1], Fei Zhu[1], Hang Yang[1], Mingjun Rao[1], Yi li[1], Huaying Liang[1], Minhui Dai[1], Ben Liu[1], Lingli Chen[1], Duoduo Han[1], Rongli Lu[1], Wenzhong Peng[1], Yan Zhang[1], Chao Song[3], Yanwei Luo[4], Pinhua Pan[1*]

© 2021. Cui Y et al. Published online at http://www.aginganddisease.org/EN/10.14336/AD.2021.1023

SUPPLEMENTARY DATA

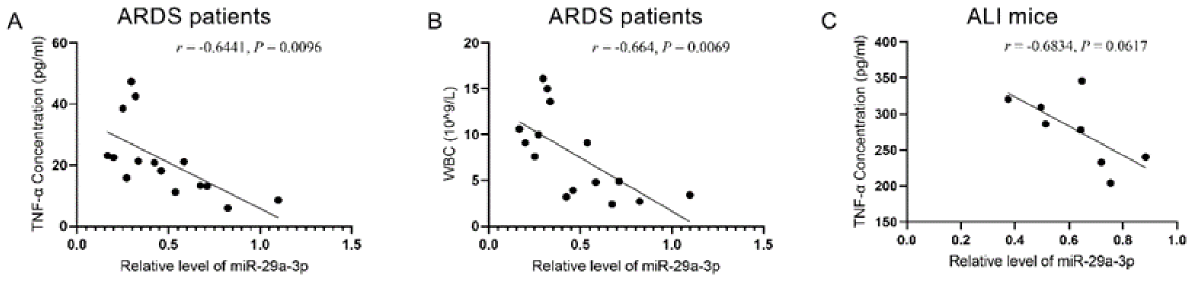

Supplementary Figure 1. MiR-29a-3p was negatively correlated with TNF-alpha plasma levels in ARDS patients and ALI model mice. (A) The correlation between miR-29a-3p expression and the TNF-α plasma levels in ARDS patients (N=15). **(B)** The correlation between miR-29a-3p expression and number of leukocytes in ARDS patients (N=15). **(C)** The correlation between miR-29a-3p expression and the TNF-α plasma levels in ALI model mice (N=8).

© 2021. Cui Y et al. Published online at http://www.aginganddisease.org/EN/10.14336/AD.2021.1023
